# The lincRNA *JUNI* regulates the stress-dependent induction of c-Jun, cellular migration and survival through the modulation of the DUSP14-JNK axis

**DOI:** 10.1038/s41388-024-03021-4

**Published:** 2024-04-02

**Authors:** Vikash Kumar, Xavier Sabaté-Cadenas, Isha Soni, Esther Stern, Carine Vias, Doron Ginsberg, Carlos Romá-Mateo, Rafael Pulido, Martin Dodel, Faraz K. Mardakheh, Alena Shkumatava, Eitan Shaulian

**Affiliations:** 1 Department of Biochemistry and Molecular Biology, Institute for Medical Research Israel-Canada, Faculty of Medicine, Hebrew University of Jerusalem, 9112102 Jerusalem, Israel; 2 Institut Curie, PSL Research University, CNRS UMR3215, INSERM U934, Paris, 75005 France; 3 The Mina and Everard Goodman, Faculty of Life Science, Bar-Ilan University, Ramat Gan, Israel; 4 Department of Physiology, Facultat de Medicina i Odontologia, Universitat de València & Fundación Instituto de Investigación Sanitaria INCLIVA, 46010 Valencia, Spain; 5 Biobizkaia Health Research Institute, Barakaldo, 48903 Spain; & Ikerbasque, The Basque Foundation for Science, 48009 Bilbao, Spain; 6 Centre for Cancer Cell and Molecular Biology, Barts Cancer Institute, Queen Mary University of London, John Vane Science Centre, Charterhouse Square, London, EC1M 6BQ UK; 7 Institute of Cell Biology, University of Edinburgh, Edinburgh, EH9 3BF UK; 8 Present Address: Department of Medicine and Life Sciences, Universitat Pompeu Fabra, 08003 Barcelona, Spain; 9 Present Address: Gene Therapy Institute, Hadassah Hebrew University Medical Center and Faculty of Medicine, Hebrew University, Jerusalem, 9112102 Israel

**Keywords:** Cell death, Cell migration

## Abstract

Cancer cells employ adaptive mechanisms to survive various stressors, including genotoxic drugs. Understanding the factors promoting survival is crucial for developing effective treatments. In this study, we unveil a previously unexplored long non-coding RNA, *JUNI* (JUN-DT, LINC01135), which is upregulated by genotoxic drugs through the activation of stress-activated MAPKs, JNK, and p38 and consequently exerts positive control over the expression of its adjacent gene product c-Jun, a well-known oncoprotein, which transduces signals to multiple transcriptional outputs. *JUNI* regulates cellular migration and has a crucial role in conferring cellular resistance to chemotherapeutic drugs or UV radiation. Depletion of *JUNI* markedly increases the sensitivity of cultured cells and spheroids to chemotherapeutic agents. We identified 57 proteins interacting with *JUNI*. The activity of one of them the MAPK phosphatase and inhibitor, DUSP14, is counteracted by *JUNI*, thereby, facilitating efficient JNK phosphorylation and c-Jun induction when cells are exposed to UV radiation. The antagonistic interplay with DUSP14 contributes not only to c-Jun induction but also augments the survival of UV-exposed cells. In summary, we introduce *JUNI* as a novel stress-inducible regulator of c-Jun, positioning it as a potential target for enhancing the sensitivity of cancer cells to chemotherapy.

## Introduction

Long noncoding RNAs (lncRNAs) have gained prominence as important contributors to the neoplastic process, marked by alterations in their expression and activity and substantiated by compelling functional experimental evidence [1–4]. They affect various cancer-related processes and can impact every stage of cancer development, including initiation, progression, and metastasis [4–6]. In particular, the participation of lncRNAs in stress responses, notably the DNA damage response (DDR), empowers cancer cells to modulate and surmount DNA damages and the consequent cellular reactions, thereby evade potential cell death. Consequently, lncRNAs assume a pivotal role in fostering cancer cell survival and conferring resistance to chemotherapy [7–10].

Rapid DNA damage responses (DDRs) are crucial for maintaining organismal well-being and are frequently compromised in cancer development [11]. Key contributors to the sensing, transduction, and repair of DNA damage include tumor suppressors like ATM, p53, and BRCA proteins [11]. Conversely, certain oncoproteins, such as the stress-responsive AP-1 member c-Jun, exhibit high sensitivity to environmental DNA-damaging agents like ultraviolet light (UV) [12]. The UV-induced phosphorylation of c-Jun N-terminal kinase (JNK) triggers JNK-dependent c-Jun phosphorylation, protein stabilization, and increased transcription through autoregulation of its expression [13–16].

The phosphorylation and activity of JNK are subject to regulation by multiple upstream kinases and phosphatases [14, 17, 18]. Notably, Dual-Specificity Phosphatases (DUSPs) play a significant role in this regulation. JNKs, activated by dual phosphorylation at threonine and tyrosine within the TPY motif, undergo dephosphorylation by DUSPs. This dephosphorylation negatively regulates JNK activity and its downstream targets, including c-Jun [17].

The broad responsiveness of c-Jun to environmental stimuli and its consequential involvement in multiple cellular processes critical for cellular homeostasis make it essential for cellular survival [19], development [20] and tumorigenicity [21]. Moreover, the ability of c-Jun to antagonize proapoptotic signaling underlies its capacity to endow cancer cells with drug resistance. Notably, its activation following treatment of melanomas with BRAF inhibitors is a primary driver for phenotype switching, rendering the cells more mesenchymal and associated with elevated drug resistance [22–24].

In this study, we identified *JUNI* as a stress-regulated lncRNA that controls c-Jun expression, cellular migration and most importantly enhances the survival of cancer cells following exposure to chemotherapeutic drugs.

## Results

### JUNI is a stress-regulated lincRNA

**Fig. 1:**
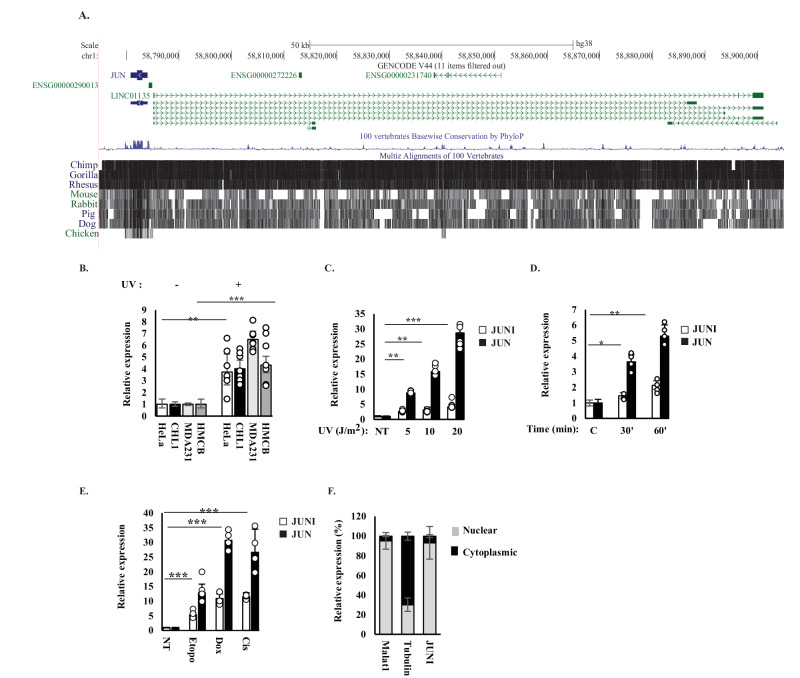
*JUNI* is a nuclear lncRNA upregulated by stress. **A** A schema of *JUNI* structure and evolutionary conservation. Green lines represent *LINC01135* isoforms (*JUNI*), black lines alignment with the indicated species. Data were obtained from the UCSC Genome Browser on Human (GRCh38/hg38). **B** Expression of *JUNI* in HeLa, CHL1, MDA-MB-231 and HMCB cells before and 4 h after exposure to 20 (Hela) or 30 (All other cell lines) J/m^2^ UV. GAPDH was used for normalization in this experiment and in all the following qPCR experiments. **C** HeLa cells were irradiated with the indicated doses of UV and harvested 4 h after irradiation. *JUNI* and *JUN* RNA levels (white and black bars, respectively) were determined by RT-qPCR. **D** HeLa cells were irradiated with 20 J/m^2^ UV and harvested 30 or 60 min after irradiation. *JUNI* and *JUN* RNA levels (white and black bars, respectively) were determined by RT-qPCR. **E** HeLa cells were untreated (NT) or exposed to 5 μM of etoposide, doxorubicin or cisplatin. *JUN* and *JUNI* RNA levels (black and white bars, respectively) were determined 4 h after treatment using RT-qPCR. **F** HeLa cells were fractionated into nuclear and cytoplasmic fractions, and the abundance of *JUNI* was examined in each fraction using RT-qPCR. The gray portion of the bar represents the nuclear fraction, and black represents the cytoplasmic fraction. Tubulin and MALAT1 RNAs were used as markers for cytoplasmic and nuclear fractions, respectively. Significance is marked in this study according to the following key: *p < 0.05, **p < 0.001, ***p < 1 × 10^−5^. N in all RT-qPCR experiments was >3.

To identify genes regulated by c-Jun, we analyzed ChIP-seq data from ENCODE for genes whose transcription start sites (TSSs) are located in the vicinity of c-Jun binding sites. The long intergenic noncoding RNA *JUN-DT* or *LINC01135*, referred to hereafter as *JUNI* (for *JUN* inducer) fits this criterion. It is positioned next to the *JUN* promoter and according to ENCODE data, transcribed in the opposite orientation to *JUN*. Only 1100 bp distinguish between the two TSSs. ENCODE data predicts that *JUNI* contains five main exons and has multiple isoforms. Twenty-seven different transcript isoforms were described according to LNCipedia ranging from 213 to 6213 bases [25]. Importantly, ENCODE predicts that the first exon is shared by all, therefore, all primers to analyze JUNI’s expression as well as siRNAs to silence it, were targeted for this exon. *JUNI* is evolutionarily conserved within primates but not in rodents (Fig. 1A). The fact that c-Jun binds its own promoter and autoregulates its expression [16, 26] supported the ChIP-seq results but did not confirm any functionality for *JUNI*. Therefore, we analyzed its expression to exclude promoter leakiness. UV-driven activation of c-Jun N-terminal kinase (JNK) results in JNK-dependent c-Jun phosphorylation, transcript elevation and protein stabilization [13–15]. Hypothesizing that the expression of *JUNI* is regulated by c-Jun and given that UV radiation is a major inducer of c-Jun expression, we irradiated four different cell types with UV radiation and examined *JUNI* expression. In this study, we used a set of cell lines, including two melanoma cell lines HMCB and CHL1 (where UV is a major carcinogen), HeLa (cervical carcinoma cell line in which *JUN* expression was intensively studied) and MDA-MB-231 (breast cancer-TNBC; a cancer type in which *JUN* plays a significant role [27]). Irradiation of these cell lines with 20-30 J/m^2^ UVC resulted in three to five-fold induction of *JUNI* expression (Fig. 1B). Similar to *JUN*, the induction was dose dependent (Fig. 1C), and the rapid response to stress (Fig. 1D) as well as to serum stimulation of starved cells, identified by others [28], qualifies it as an “immediate early” lncRNA. To determine how common is the correlation between *JUNI* and *JUN* induction following exposure to drug-induced stress, HeLa cells were exposed to different chemotherapeutic drugs that induce *JUN* expression, and *JUNI* levels were monitored. Every DNA damaging treatment that induced *JUN* also induced *JUNI*, although to a lower extent (Fig. 1E). Fractionation experiments demonstrated that *JUNI* resides mainly in the nucleus (Fig. 1F) and quantitation of *JUNI*’s copy number in untreated HMCB and MBA-MD-231 cells revealed the presence of about 8 copies per cell. HeLa cells express lower amounts of 1-2 copies in untreated cells. Overall, these results suggested that *JUNI* is a stress-induced gene whose expression pattern resembles that of *JUN*, therefore, we investigated the potential existence of regulatory interactions between the two genes, especially after exposure of cells to stress.

### *JUNI* expression is controlled by MAPKs but not by c-Jun

**Fig. 2:**
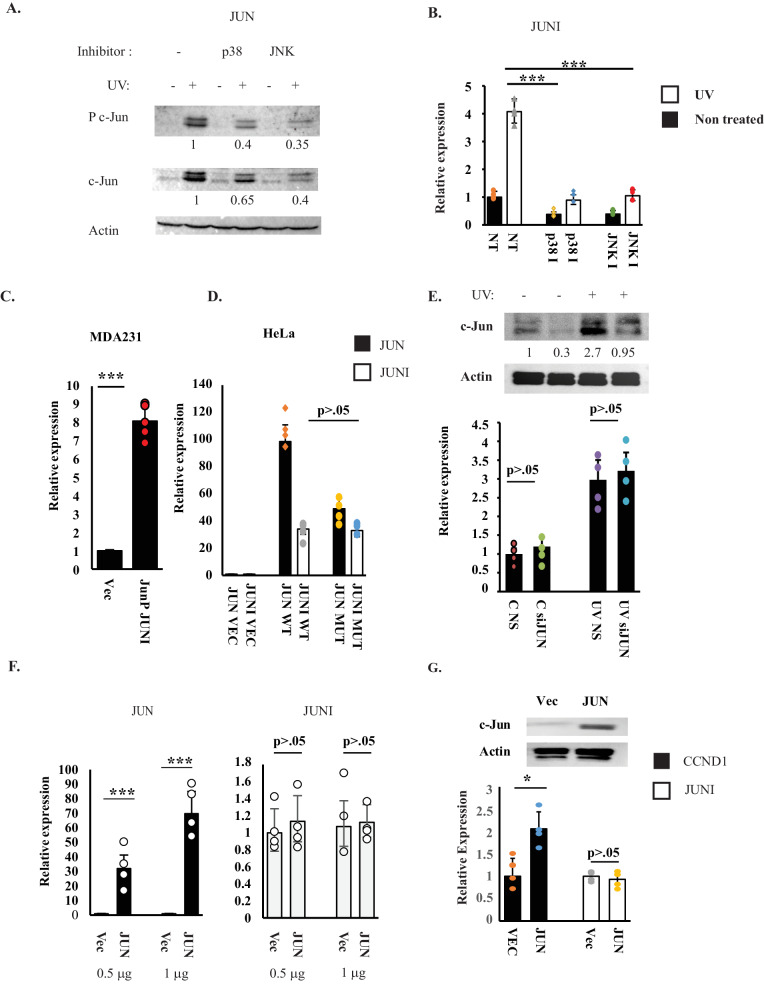
*JUNI* induction by UV is MAPK-dependent and c-Jun independent. HeLa cells were treated with 10 mM SP600125 (JNK inhibitor) or SB203580 (p38 inhibitor) 1 h prior to exposure to 20 J/m^2^ UV and harvested 2 h after exposure. Phosphorylated (Ser 63) and total c-Jun protein levels were determined using specific antibodies (**A**), and *JUNI* RNA levels were determined using RT-qPCR (**B**). Numbers under each panel of western blots indicate normalized relative quantities of the relevant protein. White bars indicate RNA from UV-exposed cells, and black bars indicate RNA from nonexposed cells. Actin was used as a loading control. **C** MDA-MB-231 cells were transfected with a vector containing the genomic sequences of the *JUN* promoter and the adjacent first exon of *JUNI* or with an empty vector. Forty-eight hours later, *JUNI* expression was monitored using RT-qPCR after DNase treatment. **D** HeLa cells were transfected with the same vector or with a vector containing mutated AP-1 binding sites in the *JUN* promoter. Levels of *JUN* (black bars) or *JUNI* (white bars) were determined using RT-qPCR. **E** HeLa cells were transfected with nonspecific (NS) or *JUN*-specific siRNAs (10 nM), irradiated with 20 J/m^2^ 48 h post transfection or not, and harvested 3.5 h later. c-Jun protein (upper panel) and *JUNI* levels (lower panel) were measured using immunoblotting and RT-qPCR. **F** HeLa cells were transfected with the indicated amounts of empty vector or c-Jun expression vector. The levels of *JUN* RNA (black bars) or *JUNI* (gray bars) were measured using RT-qPCR. **G** HeLa cells were transfected with 500 ng of empty vector or c-Jun expression vector. Cells were harvested 48 h later, and c-Jun protein levels (upper panel) or *CCND1* (black bars) and *JUNI* (white bars) RNA levels, were measured using immunoblotting and RT-qPCR, respectively. Westerns in this experiment and all the following figures *N* ≥ 3.

To define the pathways required for *JUNI* upregulation after stress exposure, we inhibited two UV-activated kinases that are known to phosphorylate/induce c-Jun, JNK and p38 using specific inhibitors SP600125 (JNK) or SB203580 (p38). Both inhibitors inhibited *JUNI* induction by UV, similar to c-Jun expression (Fig. 2A, B), suggesting that the expression of *JUNI* may be c-Jun-dependent. As the *JUN* promoter is the major regulatory element proximate to the first exon of *JUNI*, we tested whether it co-regulates *JUNI* expression. We transfected MDA-MB-231 and HeLa cells with a genomic element that contains the promoter of *JUN* flanked by 153 bases of the first exon of *JUNI* on one side and 750 bp of the 5′ UTR of *JUN* on the other side [26]. Examination of RNA extracted after DNase treatment demonstrated 8- and 34-fold higher expression of *JUNI* relative to cells transfected with an empty vector in the different cell lines (Fig. 2C and D). Thus, the genomic sequence within the *JUN* promoter acts bidirectionally, driving the expression of *JUN* on one side and *JUNI* on the other, as previously described for other lncRNAs [29]. Two AP-1 binding sites in the *JUN* promoter mediate autoregulation of *JUN* expression. Surprisingly, although mutation in one of them (see materials and methods) [19] reduced *JUN* expression relative to the promoter expressing wt AP-1 binding sites, *JUNI* expression levels did not change, suggesting that *JUNI* is not regulated by c-Jun (Fig. 2D). To directly explore the dependence of *JUNI* expression on c-Jun, specific siRNA for *JUN* was transfected into HeLa cells, the cells were UV-treated or not, and the levels of c-Jun and *JUNI* were determined (Fig. 2E). Despite three-fold suppression of c-Jun protein expression*, JUNI* levels did not decline (Fig. 2E). Reciprocal experiments supported the independence of *JUNI* from c-Jun expression. HeLa cells transfected with the *JUN* expression vector and expressing very high levels of *JUN* mRNA did not present any difference in *JUNI* expression (Fig. 2F), and unlike the induction of *CCND1*, a known c-Jun target gene [30], *JUNI* levels were not changed in *JUN*-transfected cells expressing high levels of c-Jun protein (Fig. 2G), thus proving that *JUNI* expression is independent of c-Jun.

### *JUNI* regulates c-Jun expression

**Fig. 3:**
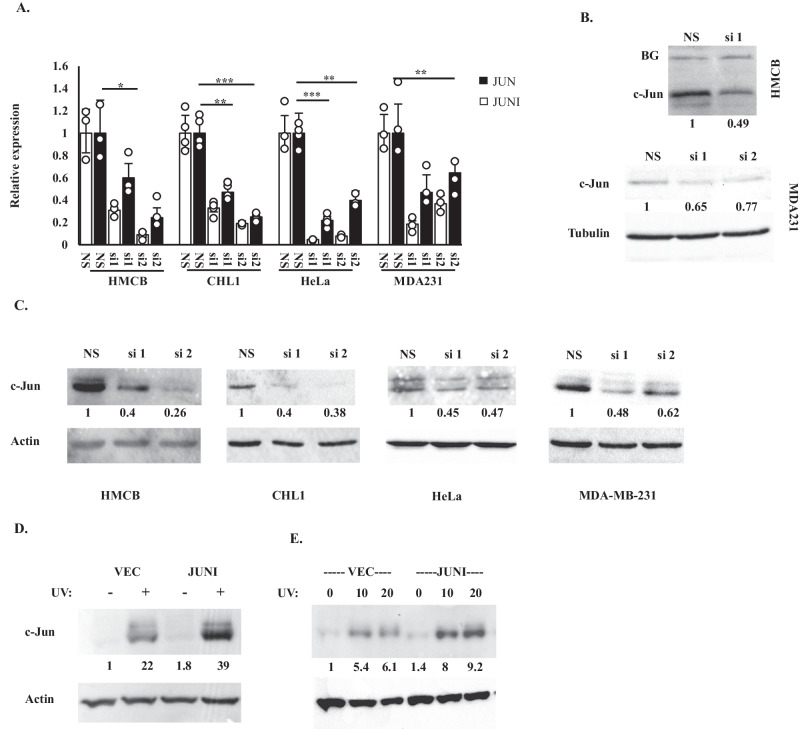
*JUNI* regulates the expression of c-Jun. **A** The indicated cells were transfected with nonspecific siRNA (NS) or two siRNAs against *JUNI* (si1: 20 nM and si2: 5 nM). The cells were harvested 24 h later, and RT-qPCR was performed to determine *JUNI* (white bars) and *JUN* (black bars) levels **B** HMCB cells (upper part) and MDA-MB-231 cells (lower part) were transfected with NS- or *JUNI*-specific siRNAs and harvested 48 h later. c-Jun protein levels were determined by immunoblotting using a specific antibody. Equal background bands (BGs) or actin were used as loading controls. **C** The indicated cells were transfected with NS- or *JUNI*-specific siRNAs and exposed to UV radiation. c-Jun protein levels were examined by immunoblotting 4–6 h after exposure using a specific antibody. Actin was used as a loading control. HeLa cells were transiently (**D**) or stably transfected (**E**) with the first exon of *JUNI* or with an empty vector. Cells were irradiated 36 h post transfection with 20 J/m^2^ (**D**) or with the indicated UV doses (**E**), and the levels of c-Jun protein were measured 4 h later as described above.

Given that many lncRNAs modulate the expression of nearby genes in a *cis* [31], we examined the effects of *JUNI* on *JUN* expression. To this end, we attempted to knock out the first exon of *JUNI* using CRISPR gene editing targeting *JUNI* with two sgRNAs flanking its first exon. However, exon 1-deleted clones could not be generated, suggesting an essential role for this gene. As the first exon is common to all isoforms, we designed two siRNAs targeting it and used them to transiently silence *JUNI* expression. Both siRNAs silenced *JUNI* and consequently *JUN* mRNA expression to a lesser degree (Fig. 3A). It is worth mentioning that cell line-specific, consistent variations in the silencing capacities of the two siRNAs were observed. Importantly, all the phenotypic effects described in this study corresponded to the silencing efficiencies of each siRNA. HMCB and MDA-MB-231 cells express high levels of c-Jun protein, which can be detected even without stress exposure. A mild reduction in c-Jun protein levels after *JUNI* silencing was detected in these cells (Fig. 3B). In contrast, a clear effect of silencing was observed in all cell lines after exposure to UV (Fig. 3C), suggesting that *JUNI* has special importance for c-Jun expression after exposure of cells to stress. Indeed, exogenous expression of relatively low levels of the first exon of *JUNI* either transiently (Fig. 3D) or stably (Fig. 3E) was sufficient to elevate c-Jun levels in cells exposed to UV radiation. Combined, our data suggest that *JUNI* can regulate *JUN* expression in *trans* and is particularly important after stress exposure.

Significant correlations between *JUNI* and *JUN* expression also occur in vivo. Examination of expression data of 32 types of tumors in the Pan-Cancer Co-Expression Analysis for the RNA-RNA interactions [32] revealed a 94% positive correlation between *JUNI* and *JUN* levels in all cancer types examined. Sixty-two percent of the positively correlated cases were statistically significant (Table 1).

**Table 1:** Correlations of *JUNI* and *JUN* co-expression in 32 cancer types.

Cancer type	Cofficient-*R*	sample size	*p*-value
Breast invasive carcinoma	0.121	1104	**5.20E-05**
Cervical squamous cell carcinoma and endocervical adenocarcinoma	0.157	306	**6.02E-03**
Adrenocortical carcinoma	0.187	79	**9.97E-02**
Bladder urothelial carcinoma	0.172	411	**4.69E-04**
Cholangiocarcinoma	0.264	36	**1.20E-01**
Colon adenocarcinoma	0.033	471	**4.69E-01**
Lymphoid neoplasm diffuse large B-cell lymphoma	0.415	48	**3.37E-03**
Esophageal Carcinoma	0.277	162	**3.58E-04**
Head and neck squamous cell carcinoma	0.146	502	**1.02E-03**
Kidney chromophobe	0.198	65	**1.14E-01**
Kidney renal clear cell carcinoma	0.094	535	**2.98E-02**
Acute myeloid leukemia	0.424	151	**5.94E-08**
Brain lower grade glioma	0.131	529	**2.56E-03**
Liver hepatocellular carcinoma	0.237	374	**3.67E-06**
Lung adenocarcinoma	0.213	526	**7.88E-07**
Lung squamous cell carcinoma	0.162	501	**2.61E-04**
Ovarian serous cystadenocarcinoma	0.222	379	**1.30E-05**
Pancreatic adenocarcinoma	0.239	178	**1.25E-03**
Pheochromocytoma and paraganglioma	0.338	183	**8.24E-02**
Prostate adenocarcinoma	0.219	499	**7.71E-01**
Rectum adenocarcinoma	0.231	167	**2.17E-01**
Sarcoma	0.308	263	**2.87E-05**
Skin cutaneous melanoma	0.123	471	**3.02E-02**
Stomach adenocarcinoma	0.253	375	**1.10E-01**
Testicular germ cell tumors	0.402	156	**2.02E-04**
Thyroid carcinoma	0.261	510	**1.49E-01**
Thymoma	0.532	119	**1.30E-12**
Uterine corpus endometrial carcinoma	0.136	548	**5.04E-18**
Uterine carcinosarcoma	0.25	56	**1.23E-01**
Uveal melanoma	0.285	80	**7.33E-01**
Mesothelioma	−0.184	86	**9.07E-02**

Data obtained from the Pan-Cancer Co-Expression Analysis for the RNA–RNA interactions [32].

### *JUNI* affects c-Jun-regulated EMT effectors and cellular migration

**Fig. 4:**
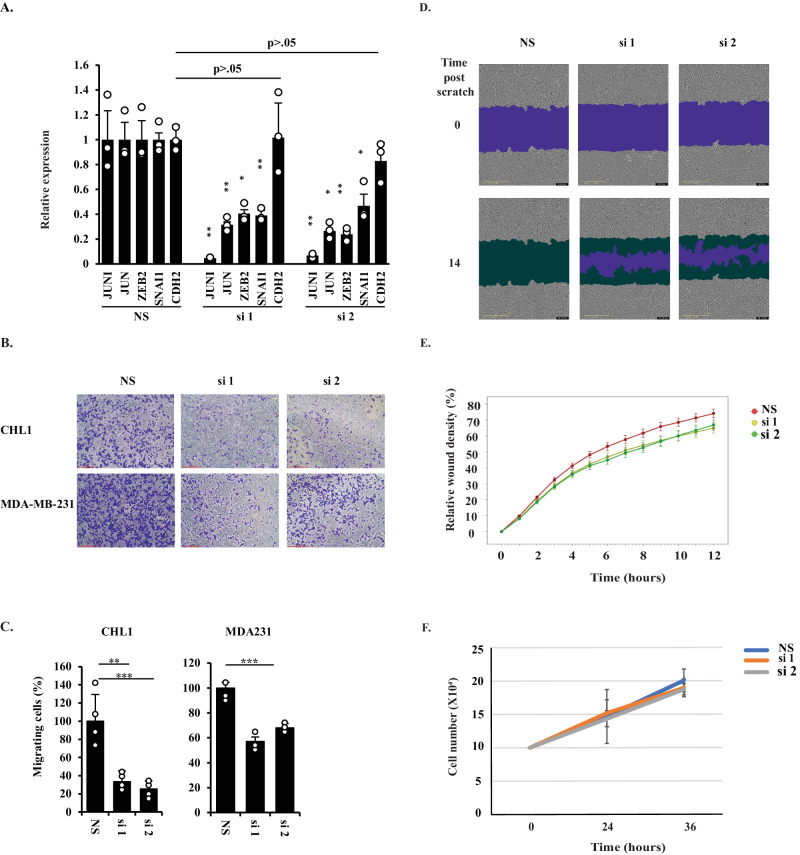
*JUNI*’s expression is essential for cellular migration. **A** CHL1 cells were transfected with the indicated siRNAs. Expression of the indicated genes was determined 24 h later using RT-qPCR and specific primers. **B** The indicated cell lines were transfected with the indicated siRNA. 5 × 10^5^ cells were seeded 24 h after transfection on 8-micron Millicell hanging insert membrane and the crossing cells were stained with methylene blue and photographed 16 later. **C** Graphic presentation of cells crossing the Millicell membrane16 hours post plating. The migration of cells transfected with NS was considered 100%. The mean plus SD is presented. N: CHL1 = 4; MDA-MB-231 = 3. **D** CHL1 cells transfected with the indicated siRNA were confluently seeded, scratched and wound closure was monitored at 1 h intervals for 12 h using the IncuCyte. Purple = Cell free wound area. Dark green= Area repopulated by migrating cells. *N* = 8. **E** Graphic representation of the ratio of the occupied area relative to the total area of the initial scratched region (relative wound density) over time. Each point is the average of wound closure in eight wells plus SD. **F** 10^5^ CHL1 cells/well were transfected with the indicated siRNAs and counted 24 and 36 h post transfection. Average + SD is presented. *N* = 3.

We examined whether *JUNI* has a regulatory impact on targets downstream of c-Jun. As c-Jun can modulate melanoma cells plasticity [23], we examined the expression of a set of known c-Jun-regulated genes in CHL1 cells 24 h after silencing *JUNI*. Interestingly, c-Jun target genes known to be involved in epithelial-mesenchymal-transition (EMT), such as ZEB2 [33] and SNAI1 [34], were downregulated by *JUNI* silencing, whereas CDH2, which is not a known target, was not (Fig. 4A). One of the potential consequences of downregulation of EMT-relevant, c-Jun targets is reduction in cellular migration as c-Jun was reported to regulate motility [35–38]. Therefore, we examined whether *JUNI*’s silencing will reduce the motility of CHL1 and MDA-MB-231 cells. *JUNI* was silenced using two different siRNAS or not and the ability of both cell types to migrate through a porous membrane was measure using transwell migration assay. As depicted in Fig. 4B, C*JUNI* silencing reduced the migration relative to control cells (NS). This suggests that *JUNI* is required for cellular migration. To further support this conclusion CHL1 cells in which *JUNI* was silenced or not were subjected to scratch analysis. Indeed, as expected, silencing of *JUNI* impaired the ability of the silenced cells to migrate and close the wound (Fig. 4D, E). To exclude the possibility that these effects are due to spontaneous reduction in cellular survival, at this early time point, *JUNI* silenced CHL1 cells were counted 24 and 36 h after transfection, the final time points of the migration assays. At this time point 6% difference in cell number was observed between JUNI expressing and *JUNI* silenced cells (Fig. 4F) clearly distinguished from the 60–70% difference observed in the transwell migration (Fig. 4C). These results demonstrate that *JUNI* can regulate the levels of genes involved in EMT and control cellular migration.

### Short-term silencing of *JUNI* sensitizes cancer cells to UV radiation and chemotherapeutic drugs

**Fig. 5:**
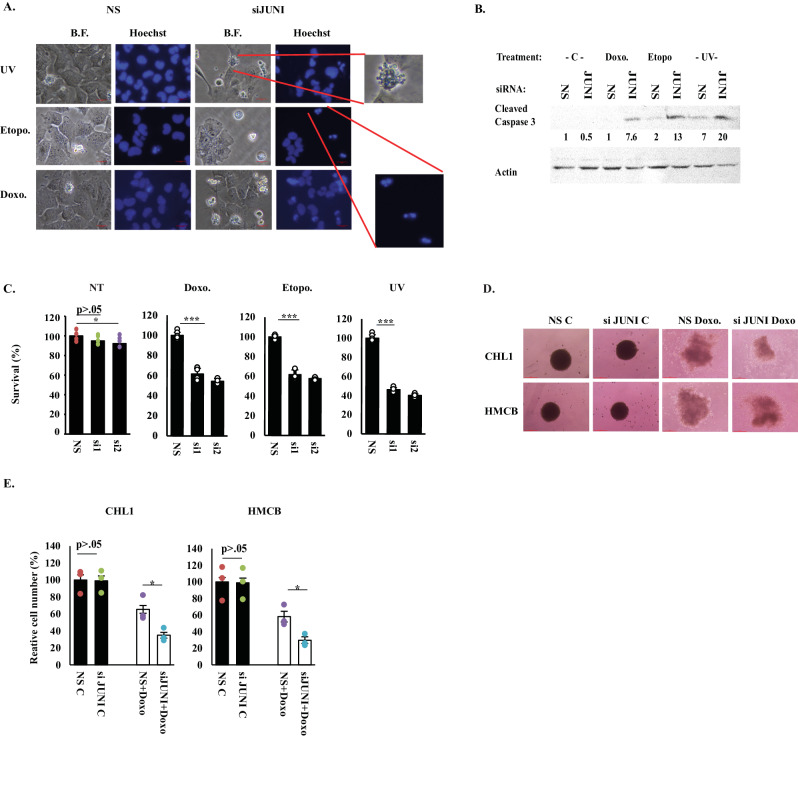
*JUNI* is essential for the survival of stress exposed cancer cells. **A** HMCB cells were transfected with the indicated siRNAs. Thirty-six hours post transfection, the cells were treated with either 20 J/m^2^ UVC, 10 μM etoposide or 5 μM doxorubicin. 12 h later the cells were stained with Hoechst and photographed. Characteristic morphologies of apoptotic cells are blown up. **B** HMCB cells were transfected with the indicated siRNA and treated 36 h later with 5 μM doxorubicin, 25 μM etoposide or 20 J/m^2^ UVC and harvested 12-18 h later to determine the presence of cleaved caspase 3 using specific antibody. Actin was used as a loading control and relative levels are indicated. **C** HMCB cells were transfected with the indicated siRNAs and treated 36 h later with 1μM doxorubicin, 5μM etoposide or 15 J/m^2^ UV. XTT was measured 20 h later. Survival of NS -transfected cells was considered 100% in this and in the following XTT experiments. NT; cell death in non-treated cells. N in all XTT experiments is >3. Spheroids of the indicated cell lines were exposed to 1–2 μM doxorubicin, photographed 5–8 days later (**D**), collected, trypsinized and counted (**E**).

The induction of *JUNI* by DNA damaging agents (Fig. 1) suggests its potential role in the cellular response to DNA damage. Given the established association of EMT with chemoresistance [39, 40] and considering the impact of *JUNI* on EMT-relevant genes, we aimed to investigate the significance of *JUNI* in the survival of cells exposed to UV irradiation or chemotherapeutic drugs. To this end *JUNI* was silenced in HMCB, CHL-1 and MDA-MB-231 cells, and 36–40 h later, the cells were either UV irradiated or exposed to the chemotherapeutic drugs etoposide or doxorubicin. Cell survival was determined microscopically and using XTT cell viability assay. Silencing of *JUNI* in all cell lines sensitized them to UV-induced stress and chemotherapeutic drugs (Fig. 5A, B and Fig. S1). Apoptotic cells exhibiting blebbing membranes and nuclear fragmentation were clearly observed (Fig. 5A). Moreover, all treatments induced higher levels of cleaved caspase 3 in *JUNI*-silenced cells (Fig. 5B). Measurement of survival using XTT revealed that exposure to UV radiation and the administered drugs led to a 30 to 65% decrease in the survival of *JUNI*-silenced cells compared to cells transfected with control siRNA (Figs. 5C and S1). Cell death in *JUNI-*silenced cells not treated with drugs averaged around seven to eight percent in all cell lines (Figs. 5C and S1, NT), suggesting that the drugs effect is specific. To assess the impact of *JUNI* silencing on the sensitivity of cancer cells to chemotherapeutic drugs in another model, spheroids of CHL1 and HMCB cells were generated, exposed to doxorubicin, and analyzed for survival 5-8 days later. As illustrated in Fig. 5D, E, the survival of *JUNI*-silenced cells was diminished by 50%, further supporting the protective role of *JUNI* in cells exposed to DNA damage. Overall, we suggest that *JUNI* is indispensable for the survival of stress-exposed cancer cells.

### Antagonistic interactions of *JUNI* with DUSP14 mediate c-Jun induction by UV and cellular survival

**Fig. 6:**
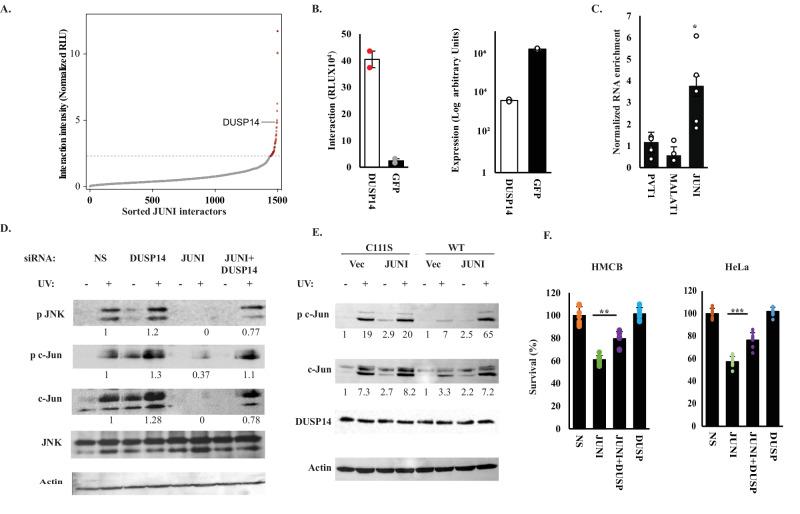
*JUNI* interacts with DUSP14 and antagonizes its activity. **A** Normalized interaction intensities of *JUNI* and potential protein interactors, averaged from two incPRINT biological replicates, sorted in increasing order. The horizontal dotted line represents the interaction intensity cutoff used for the classification of *JUNI* interactors. Red dots are *JUNI*-interacting proteins; gray dots are proteins that do not bind to *JUNI*. See the ‘Methods’ section for data normalization. RLU are relative light units. **B** Intensities of *JUNI* interactions with DUSP14 and GFP were obtained from two biological replicates of the incPRINT screen. On the left, interaction intensities detected between *JUNI* and the indicated proteins. On the right, the expression level of each protein was measured using ELISA. **C** HeLa cells were transfected with DUSP14 or GFP and UV crosslinking immunoprecipitation was performed. The ratio of RNAs coprecipitated with DUSP14 relative to RNAs in the whole cell extract was further normalized to the ratio obtained after GFP precipitation. Enrichment of the indicated lncRNAs is depicted. **D** HMCB cells were transfected with the indicated siRNAs, irradiated with 30 J/m^2^ 48 h later and harvested 5 h post irradiation. Levels of the indicated proteins were measured by immunoblotting using specific antibodies as described before. **E** HMCB cells were transfected with the indicated plasmids and 24 h later irradiated or not with 25 J/m^2^ UV. Cells were harvested 4 h later and the levels of the indicated proteins were measured using specific antibodies. DUSP14 was measured using HA antibody. **F** HMCB and HeLa cells were transfected with the below indicated siRNAs and UV irradiated 40 h later or not. Irradiated cells were harvested 12 h after irradiation and viability was determined using XTT.

To explore the identity of protein interactors that may affect the ability of *JUNI* to regulate c-Jun expression and affect cell death, we identified proteins interacting with *JUNI* by applying the incPRINT screen [41]. Using the shortest yet stable isoform of *JUNI* that contained the *JUN*-affecting sequences as RNA bait, we identified 57 *JUNI*-interacting proteins (Fig. 6A). Analyses of their cellular functions revealed enrichment of proteins involved in various fundamental, basic aspects of cellular wellbeing, such as splicing, ribosome biosynthesis, and mitosis (Table S1). Interestingly, c-Jun itself does not interact with *JUNI* (Table S1, Normalized luciferase intensity MS2, RLU = 0.44). By contrast, the dual specificity protein phosphatase 14 (DUSP14; also known as MKP6) was one of the highly rated interacting proteins with *JUNI* (Fig. 6A, B). DUSP14 can directly dephosphorylate JNK, p38 and ERK or indirectly limit the activation of TAK1 a MAP3K which is essential for c-Jun expression [42, 43]. We validated the interaction between DUSP14 and *JUNI* in HeLa cells by crosslinking and immunoprecipitation (CLIP) analysis. The enrichment of endogenous *JUNI* following immunoprecipitation with transfected DUSP14 or GFP was measured whereas specificity was determined by comparison of *JUNI* enrichment to other unrelated nuclear lncRNAs *MALAT1* and *PVT1*. These experiments demonstrated a specific association of *JUNI* with DUSP14 (Fig. 6C). As *JUNI* is required for efficient c-Jun upregulation after UV exposure, and DUSP14 inhibits JNK, a stress-activated, positive regulator of *JUN* transcription and protein expression [14, 15], we hypothesized that *JUNI* antagonizes DUSP14 activity to enable full-scale c-Jun induction post-stress exposure and that this antagonism may have a role in other *JUNI*-dependent activities discovered in this study. The potential *JUNI*-DUSP14 antagonism was examined at 2 levels. Biochemically, we sought to test if *JUNI* enables efficient JNK and c-Jun phosphorylation, thus resulting in c-Jun induction post UV exposure and at the cellular level, we examined whether *JUNI*-DUSP14 antagonism is reflected by effects on cellular survival.

To explore the effects on c-Jun, we silenced DUSP14, *JUNI* or both in HMCB cells that were later exposed to UV radiation and JNK activation, c-Jun phosphorylation and its total level were determined. Indeed, silencing *JUNI* inhibited JNK phosphorylation (pThr183/pTyr185) in UV-treated cells to a nearly undetectable level and consequently Ser63 phosphorylation of c-Jun, therefore, prevented its induction by UV (Fig. 6D). Co-silencing of DUSP14 together with *JUNI* restored JNK and c-Jun phosphorylation to levels slightly lower than those observed in non-silenced cells. To determine if the effect of *JUNI* on c-Jun induction is mediated by DUSP14 phosphatase activity we transfected HMCB cells with wt or phosphatase dead (C111S) DUSP14 together with *JUNI* expression vector or an empty one. Cells were irradiated 24 h post transfection and the phosphorylation as well total levels of c-Jun were monitored (Fig. 6E). As depicted in Fig. 6E, *JUNI* considerably elevated c-Jun phosphorylation and expression only in cells transfected with phosphatase active DUSP14. These data suggest that *JUNI* regulates JNK phosphorylation and c-Jun levels by antagonizing DUSP14 activity.

To assess the importance of DUSP14 in *JUNI*-dependent effects on the survival of stress-exposed cells, *JUNI*, DUSP14, or both were silenced in HMCB and HeLa cells that were later exposed to UV, and cell survival was measured (Fig. 6F). Consistent with the previously indicated results (Fig. 5), *JUNI*-silenced cells were 40% more sensitive to radiation than non-silenced cells (Fig. 6F). DUSP14 silencing did not significantly improve the survival of irradiated cells expressing normal levels of *JUNI*. Remarkably, it improved the survival of *JUNI*-silenced cells by 29 and 33% in HMCB and HeLa cells, respectively (Fig. 6F), suggesting that *JUNI*’S expression is important for antagonizing DUSP14 in order to improve the survival of stress-exposed cells. Nevertheless, it should be mentioned that the negative effect of *JUNI* silencing on the survival of stressed cells was not fully rescued by silencing of DUSP14, suggesting the presence of additional targets.

## Discussion

Overall, we discovered a novel lncRNA that plays fundamental roles in cellular survival after exposure of cancer cells to stress. *JUNI*’s expression itself is stress regulated. The known nonspecific inhibition of transcription initiation [44] and elongation [45] observed after UV exposure, and the resemblance of its to its adjacent gene, *JUN*, support the specificity of *JUNI* induction under stress. *JUNI* demonstrates a rapid response to UV exposure, consistent with its role as an inducer of c-Jun, an “immediate early protein.” Its rapid induction also in serum starved cells after complementation of serum [28], similar to c-Jun itself, also support its activity as “immediate early lncRNA”. The observation that *JUNI’s* induction does not necessitate c-Jun expression underscores the unidirectional impact as a regulator of c-Jun without reliance on its regulated gene. *JUNI*’s induction after stress exposure requires activation of JNK and p38, thereafter, *JUNI* binds and antagonizes DUSP14, a negative regulator of these kinases, to enable their full activation after stress exposure. Thus, we demonstrate a positive feedback loop in the activation of these kinases. This finding aligns with existing studies indicating the regulatory role of lncRNAs in the JNK pathway [46–49]. In fact, the lncRNA TCONS_00145741 activates JNK phosphorylation by inhibiting its interactions with DUSP6 in thrombin treated microglia cells [50]. We demonstrate that *JUNI* alleviates the inhibitory, phosphatase-dependent, effects imposed by DUSP14 of the JNK pathway, following cellular exposure to stress. While our work reveals a novel mechanism of c-Jun activation through the inhibition of the JNK inhibitor, DUSP14, in trans after stress exposure, it does not necessarily preclude additional effects of *JUNI* on c-Jun expression in untreated cells through interaction with other proteins, *in cis*.

This study attributes two crucial biological functions to *JUNI*: the regulation of cellular migration and, more significantly, its essential role in evading cell death following the exposure of cancer cells to UV and chemotherapeutic drugs. Both traits, migration and resistance to drugs, observed after *JUNI* silencing were previously associated with the activity of the JNK-c-Jun pathway. For example, JNK and c-Jun were previously reported to regulate cellular migration under different cellular settings [35–38, 47] and c-Jun was reported to inhibit apoptosis in cells exposed to DNA damages. In vitro, phosphorylation mutant c-Jun (S63A,S73A) which cannot be activated by JNK, markedly inhibited the activation of AP-1-driven transcription and greatly increased the cytotoxic effects of DNA-damaging agents associated with enhanced apoptosis [51]. ChIP-seq analysis of BT474 cells treated with cisplatin suggested that c-Jun in cooperation with ATF2 bind to regulatory elements and activates an array of DNA repair genes [52]. Moreover, liver tumors induced by carcinogen treatment (Diethylnitrosamine) were not formed in mice deleted of c-Jun, whose role in the liver is mainly in the prevention of apoptosis [21].

JNK activity is required for the induction of cell death post UV exposure [53]. Therefore, the ability of DUSP14 silencing to rescue from the cell death upon *JUNI* silencing is surprising. However, recent studies discovered a novel facet of JNK, demonstrating its cellular protective roles [54]. Three potential mechanisms may account for the rescue. Firstly, DUSP14 inhibition increases the activity of protective proteins that are negatively regulated by it. For example, NFκB is downstream of TAK1-TAB1 proteins which are negatively regulated by DUSP14 [43]. Secondly, low constitutive JNK activation may induce autophagy which exerts protective activity [54, 55]. Finally, the indirect suppression of genes involved in EMT may lead to a less mesenchymal phenotype reflected also by reduced motility. Given that EMT is associated with increased drug resistance [39, 40], it is possible that the mesenchymal to epithelial transition (MET) process occurring in these cells after silencing of *JUNI* sensitizes the cells to chemotherapeutic drugs as well as impair their migration. In summary, this study illustrates the requirement of *JUNI* for the survival of cancer cells following exposure to stress and proposes its potential as a target for chemo-sensitization.

## Materials and methods

### Cell culture

CHL-1, HeLa, MDA-MB 231, HACAT and HMCB cells were procured from ATCC. The first 4 were cultured in DMEM with 10% FBS, whereas HMCB cells were cultured in EMEM. All cells were grown with 10% FBS and cultured at 37 °C with 5% CO_2_. Cells were routinely monitored for mycoplasma contamination.

### Transfection, treatments, and inhibitors

Plasmids were transfected into 40–50% confluent cells using PloyJet reagent (SignaGen Lab MD, USA). siRNAs were transfected with the siRNA transfection reagent INTERFERin® (Polyplus, Illkirch – France). The siRNA concentrations used in this study ranged between 5 and 20 nm. SP600125 (JNK) or SB203580 (p38) inhibitors (10 µM) were added 1 h prior to UV irradiation. Doxorubicin, etoposide and cisplatin were used at doses of 0.5–5 µM. XTT viability assays (Abcam, Cambridge UK) were used according to the manufacturer’s instructions.

### RNA Extraction and real-time PCR

RNA was isolated manually using TRI Reagent® (MRC, OH, USA). cDNA was prepared from total RNA (1 µg) using Quantabio, and a qScript^TM^ cDNA synthesis kit and PerfeCTa SYBR Green SuperMix were used for qPCR according to the manufacturer’s instructions (Quantabio, MA, USA). To prepare DNA-free RNA samples, RNA was treated with PerfeCTa® DNase I according to the manufacturer’s instructions (Quantabio, MA, USA). A StepOne Plus Real-Time PCR apparatus with StepOne Software v2.3 was used for analysis (Applied Biosystems, MA USA). Each experiment was performed in multiple technical duplications (*n* > 4) with in 4 biological repeats. Relative mRNA expression was analyzed according to the 2^(−ΔΔCt)^ calculation method.

### Calculation of JUNI copy number

RNA was extracted from 1.5 × 10^5^ HMCB and MDA-MB-231 cells, cDNA was prepared and qPCR performed with primers aimed at the first exon of *JUNI* as described above. To calculated the total amount of *JUNI*/well CT values of *JUNI* RNA obtained from both cell lines were compared to calibration curve of CT values (y) of known amounts of *JUNI* expressing plasmid (X). Number of copies/well (NCW) was calculated using the following equation: NCW = (ng × [6.022 × 10^23^])/(length × [1 × 10^9^] × 650). Number of copies/cell (NCC) was calculated by the following formula: NCC = NCW*dilution factor from total RNA to cDNA in a single well/number of cells from which the RNA was extracted. Taken into account 20-30% loss of RNA in extraction and RTqPCR process the number of copies presented is the minimal copy number per cell.

### Antibodies, immunoblotting and proteins quantification

The primary antibodies used were c-Jun (CST #60A8 1:1000), Ser-63-p-JUN (CST #9261 1:500), actin (CST #3700S 1:5000), GAPDH (CST #97166 1:5000), P-JNK (CST #9251 1:500), total JNK (#9252, #9258 1:1000), cleaved Caspase-3 Asp175 (5A1E #96641:1000) and anti-FLAG M2 antibody (Sigma, #F1804). HRP-conjugated secondary antibody was used at a dilution of 1:5000 (Jackson Laboratories, USA). Immunoblotting was performed using standard protocol. Proteins were detected and quantitated using Bio-Rad’s chemiDoc imagers. Representative images are presented. Proteins quantities normalized to loading controls are presented below each relevant blot. In all cases *N* ≥ 3.

### Spheroid formation

2–3k cells were seeded using hanging drop method, 5–7 days later spheroids were transferred to ultra low-attachment U-bottom 96 well plate and treated with 1–2 µM of doxorubicin for 5–8 days. Images were acquired at ×10 magnification, spheroids were trypsinized and total cells number was counted using Trypan blue dye, *N* = 3.

### Cell migration

Fifteen thousand CHL1 cells/well were seeded in a 96-well dish (Sartorius IncuCyte Imagelock Plate) and transfected with the desired siRNA and 24 h later 24 h later. Wounds were made 24 h post transfection using wound making tool to create an equal scratch and IncuCyte live cell imaging system (Sartorius) was used to measure wound closure for 12 h, taking pictures at 1 h intervals. *N* = 8

### Microscopy

Images were captured at ×40 magnification using an inverted Nikon Eclipse Ts2R microscope. Scale bars are presented.

### Plasmids and cloning

DUSP14-HIS was a kind gift from M. Saleh [56] pRK5 HA-DUSP14 plasmid (HA tag N-terminal) was obtained by PCR amplification from pOTB7 DUSP14 plasmid (HGMP MRC geneservice, IMAGE cDNA clone 2819474) and subcloning into pRK5 plasmid containing HA sequence. pRK5 HA-DUSP14 C111S was obtained by PCR oligonucleotide site-directed mutagenesis. To generate the *JUNI*-MS2x10 construct used in incPRINT, a 938 bp fragment of the *JUNI* mature RNA was cloned into 10xMS2 vector [41] using a gBlocks Gene Fragment containing Cla1 and Hind3 restriction sites (IDT, NJ, USA). Exon1 of *JUNI* was cloned into pEFA1 CMV puro/GFP vector using a gBlocks gene fragment containing EcoR1 and BAMH1 restriction enzymes. The genomic element that contains the promoter of *JUN* flanked by 153 bases of the first exon of *JUNI* on one side and 750 bp of the 5′ UTR of *JUN* on the other side as well as its mutant versions were previously described [26]. The AP-1 binding site at positions -72- -63 of the *JUN* promoter was mutated from 5′-GTGACATCAT-3′ to 5′-CATCCACCAT-3′ as previously described [19].

### Crosslinking and Immunoprecipitation (CLIP)

Ten million HeLa cells were transiently transfected with mammalian expression vectors expressing His-DUSP14 or GFP as a control using Lipofectamine 2000 (Thermo Fisher) according to the manufacturer’s instructions. The next day, the cells were washed with PBS and irradiated once on ice with 150 mJ/cm^2^ UV light (254 nm) in ice-cold PBS using a Hoefer Scientific UV Crosslinker. Cells were then pelleted and lysed in 1 ml of lysis buffer (50 mM Tris-HCl pH 7.4, 100 mM NaCl, 1% Igepal CA-630, 0.1% SDS, 0.5% sodium deoxycholate, supplemented with protease inhibitors), sonicated, cleared, and adjusted to a protein concentration of 1 mg/ml. RNA was then partially digested with 0.2 U/ml RNase I for 3 min before immunoprecipitation with 10 μL of anti-His-tag antibody (Cell Signaling) pre-conjugated to 50 μL protein G Dynabeads (Thermo Fisher) for 1 h. The beads were then washed 5 times with lysis buffer before the bound RNAs were eluted off by Proteinase K (Thermo Fisher) digestion for 1 h at 65 °C. The RNA was then extracted using TRIzol (Thermo Fisher) and subjected to qPCR analysis with qPCR primers against *JUNI*, *MALAT1*, and *PVT1*.

### IncPRINT

The incPRINT protocol and analysis were previously described [41]. In this study, the bait was a 938 bp fragment of the *JUNI* mature RNA cloned into the 10xMS2 vector. The experiment was performed twice. The threshold for significant interaction was set at a relative luciferase activity (protein versus background) 2.2 or higher.

### Primers and siRNA

#### PCR primers

**Table:** 

Gene	Forward	Reverse
GAPDH	TCGACAGTCAGCCGCATCTTCTTT	ACCAAATCCGTTGACTCCGACCTT
JUN	CCCCAAGATCCTGAAACAGA	CCGTTGCTGGACTGGATTAT
JUNI	CGGACACTCGCATAAAGTCA	AGCGCTTCCTAGAGGCTACC
Zeb2	CAAGAGGCGCAAACAAGCC	GGTTGGCAATACCGTCATCC
SNAI1	CTGGGTGCCCTCAAGATGCA	CCGGACATGGCCTTGTAGCA
DUSP14	GAGGCGTACAACTGGGTGAA	CCCCAGTAAGGCATCAGGT
CDH2	TGTCGGTGACAAAGCCCCTG	AGGGCATTGGGATCGTCAGC
MALAT1	GACGGAGGTTGAGATGAAGC	ATTCGGGGCTCTGTAGTCCT
PVT1	TGGAATGTAAGACCCCGACTCT	GATGGCTGTATGTGCCAAGGT
CCND1	GAGGAAGAGGAGGAGGAGGA	GAGATGGAAGGGGGAAAGAG

#### siRNA oligonucleotides

**Table:** 

JUNI si1	GACACUCGCAUAAAGUCACGCAGUA
JUNI si2	GGUAGAACCUGGACUUCAAAUCUCU
JUN	ACUCAUGCUAACGCAGCAGUU
DUSP14	GGCAUCACCUGCAUUGUUAAUGCTA

### Statistical analysis

All the data presented as normalized means ± SD. One tailed Wilcoxon matched pairs test was used to determine significance. *P* < 0.05 were considered as statistically significance. Significance is marked in this study according to the following key: *p < 0.05; **p < 0.001, ***p < 1 × 10^-5^.

### Supplementary information

Supplemental Material

Supplement Figure 1
