# Supplementary material for: The lincRNA JUNI regulates the stress-dependent induction of c-Jun, cellular migration and survival through the modulation of the DUSP14-JNK axis
Source: Oncogene. 2024 Apr 2;43(21):1608–19. doi: 10.1038/s41388-024-03021-4 (PMC11108773; doi:10.1038/s41388-024-03021-4)
Supplement: Supplementary file 1 — Supplemental Material [file 41388_2024_3021_MOESM1_ESM.docx]

**Supplementary Data**

**Figure legend**

Fig. S1. Silencing of *JUN*I sensitizes cancer cells to stress-induced cell death.

A. CHL1 cells transfected with the indicated siRNA were left untreated (NT) or treated 38 h later with 30 J/m2 UV, 1μM doxorubicin or 5μM etoposide. XTT was measured 20 h later. B. MDA-MB-231 cells were transfected with the indicated siRNA and 36 h later were treated with 10J/m^2^UV, 1μM doxorubicin or 5μM etoposide, harvested 12h after UV exposure and 20h after drugs treatment and XTT levels were measured. C. HeLa cells were transfected with the indicated siRNA and survival using XTT was measured 56 h later.

In all cases survival of NS transfected cells was considered as 100%. Mean +SD is presented. N>3 in all cases.

**Table S1**.

| Protein name | Ensembl Gene ID | Luciferase intensity MS2, replicate 1, RLU | Luciferase intensity MS2, replicate 2, RLU | Average luciferase intensity MS2, RLU | ELISA MS2, replicate 1, RLU | ELISA MS2, replicate 2, RLU | Average ELISA MS2, RLU |
| --- | --- | --- | --- | --- | --- | --- | --- |
| A1CF | ENSG00000148584 | 259740 | 126240 | 192990 | 4741 | 4111 | 4426 |
| AHNAK | ENSG00000124942 | 480814 | 218036 | 349425 | 1203397 | 1040775 | 1122086 |
| AKAP8 | ENSG00000105127 | 151640 | 233480 | 192560 | 2186840 | 2201910 | 2194375 |
| ASS1 | ENSG00000130707 | 193370 | 309489 | 251429.5 | 1112908 | 1439029 | 1275969 |
| CEBPE | ENSG00000092067 | 381702 | 260471 | 321086.5 | 326011 | 267012 | 296512 |
| CNOT1 | ENSG00000125107 | 181542 | 405255 | 293398.5 | 2596 | 3046 | 2821 |
| CPEB2 | ENSG00000283087; ENSG00000137449 | 210656 | 182521 | 196588.5 | 5703 | 4072 | 4887.5 |
| CUL3 | ENSG00000036257 | 203189 | 180887 | 192038 | 26719 | 13016 | 19867.5 |
| DAZ3 | ENSG00000187191 | 141119 | 464918 | 303018.5 | 3180 | 3100 | 3140 |
| DAZAP1 | ENSG00000071626 | 387866 | 362351 | 375108.5 | 7063 | 8334 | 7698.5 |
| DDX49 | ENSG00000105671 | 123920 | 277496 | 200708 | 6332 | 6262 | 6297 |
| DLX2 | ENSG00000115844 | 67295 | 472068 | 269681.5 | 1E+07 | 1.1E+07 | 1.1E+07 |
| DUSP14 | ENSG00000275932; ENSG00000276023 | 436218 | 373625 | 404921.5 | 4278 | 3228 | 3753 |
| ELK4 | ENSG00000158711 | 314794 | 260644 | 287719 | 12607 | 10407 | 11507 |
| FAM171B | ENSG00000144369 | 256861 | 207242 | 232051.5 | 301231 | 219589 | 260410 |
| GRN | ENSG00000030582 | 180218 | 253447 | 216832.5 | 6252 | 5922 | 6087 |
| HEATR1 | ENSG00000119285 | 176319 | 271520 | 223919.5 | 3708 | 3718 | 3713 |
| HMGB1 | ENSG00000189403 | 587720 | 73132 | 330426 | 3105948 | 4521523 | 3813736 |
| HOXD10 | ENSG00000128710 | 70948 | 319521 | 195234.5 | 277745 | 214969 | 246357 |
| IFI16 | ENSG00000163565 | 119525 | 293771 | 206648 | 17410 | 33808 | 25609 |
| LARP7 | ENSG00000174720 | 308007 | 72410 | 190208.5 | 13791 | 7403 | 10597 |
| LSM6 | ENSG00000164167 | 389184 | 105810 | 247497 | 3868 | 4188 | 4028 |
| LTA4H | ENSG00000111144 | 303811 | 497834 | 400822.5 | 2890 | 3170 | 3030 |
| MRPL44 | ENSG00000135900 | 21902 | 389100 | 205501 | 1106 | 1336 | 1221 |
| NKRF | ENSG00000186416 | 222208 | 265175 | 243691.5 | 106458 | 149448 | 127953 |
| NOP16 | ENSG00000048162 | 188893 | 247511 | 218202 | 7621 | 12026 | 9823.5 |
| NPM3 | ENSG00000107833 | 193141 | 179061 | 186101 | 12686 | 16012 | 14349 |
| NUSAP1 | ENSG00000137804 | 214907 | 286986 | 250946.5 | 3881 | 4141 | 4011 |
| PLEKHA4 | ENSG00000105559 | 259247 | 128383 | 193815 | 1.4E+07 | 1E+07 | 1.2E+07 |
| PPHLN1 | ENSG00000134283 | 57771 | 319285 | 188528 | 66024 | 5128 | 35576 |
| PRDM5 | ENSG00000138738 | 178629 | 196205 | 187417 | 1972394 | 1725062 | 1848728 |
| PRDM7 * | ENSG00000126856 | 948950 | 1010725 | 979837.5 | 285025 | 341225 | 313125 |
| RBM19 | ENSG00000122965 | 215664 | 333668 | 274666 | 6588 | 7048 | 6818 |
| RBM25 | ENSG00000119707 | 114271 | 274366 | 194318.5 | 1836 | 1235 | 1535.5 |
| RBM42 | ENSG00000126254 | 119091 | 271117 | 195104 | 3050 | 4371 | 3710.5 |
| RBM45 | ENSG00000155636 | 172039 | 233339 | 202689 | 9585 | 9365 | 9475 |
| RBM47 | ENSG00000163694 | 230110 | 155949 | 193029.5 | 32701 | 49328 | 41014.5 |
| RBMS2 | ENSG00000076067 | 193852 | 219307 | 206579.5 | 23351 | 4791 | 14071 |
| RNMTL1 | ENSG00000171861 | 166356 | 277872 | 222114 | 505921 | 540941 | 523431 |
| RPL37A | ENSG00000197756 | 176670 | 213958 | 195314 | 5553 | 5313 | 5433 |
| SALL2 | ENSG00000165821 | 156438 | 224770 | 190604 | 279058 | 507479 | 393269 |
| SF3A1* | ENSG00000099995 | 601171 | 1087796 | 844483.5 | 7178 | 8870 | 8024 |
| SFPQ | ENSG00000116560 | 170842 | 246047 | 208444.5 | 3696 | 4116 | 3906 |
| SNRPB | ENSG00000125835 | 298918 | 286726 | 292822 | 7991 | 9763 | 8877 |
| SOX5 | ENSG00000134532 | 247028 | 171986 | 209507 | 6745261 | 4810924 | 5778093 |
| SP140 | ENSG00000079263 | 181876 | 187957 | 184916.5 | 5103076 | 5595590 | 5349333 |
| STAU2 | ENSG00000040341 | 365232 | 73811 | 219521.5 | 3018 | 2668 | 2843 |
| TAX1BP1 | ENSG00000106052 | 142070 | 245100 | 193585 | 12816 | 18717 | 15766.5 |
| TBRG4 | ENSG00000136270 | 129390 | 612648 | 371019 | 12424 | 14517 | 13470.5 |
| TDRD10 | ENSG00000163239 | 260221 | 305516 | 282868.5 | 6750 | 7531 | 7140.5 |
| ZCCHC24 | ENSG00000165424 | 143621 | 231274 | 187447.5 | 57616 | 76611 | 67113.5 |
| ZCCHC7 * | ENSG00000147905 | 678873 | 158929 | 418901 | 2660 | 2680 | 2670 |
| ZDHHC19 | ENSG00000163958 | 359274 | 191543 | 275408.5 | 21617 | 32321 | 26969 |
| ZNF281 | ENSG00000162702 | 141695 | 272538 | 207116.5 | 693647 | 501243 | 597445 |
| ZNF321 * | ENSG00000213801 | 853439 | 827937 | 840688 | 29267 | 34769 | 32018 |
| ZNF596 | ENSG00000172748 | 158222 | 209203 | 183712.5 | 9187 | 9958 | 9572.5 |
| ZNF641 | ENSG00000167528 | 581458 | 66504 | 323981 | 29720 | 41825 | 35772.5 |
|  |  |  |  |  |  |  |  |

**Table S1*:*** protein identified by the incPRINT to interact with *JUNI*

A list of protein coding genes whose products interact with *JUNI* in the incPRINT screen. Luciferase results represent intensity and ELISA reading protein abundance. Results of two replicas are depicted. Interacting proteins with higher scores than DUSP14 were labeled with *.
