# Supplementary figures and images for: The lincRNA JUNI regulates the stress-dependent induction of c-Jun, cellular migration and survival through the modulation of the DUSP14-JNK axis
Source: Oncogene. 2024 Apr 2;43(21):1608–19. doi: 10.1038/s41388-024-03021-4 (PMC11108773; doi:10.1038/s41388-024-03021-4)

A.

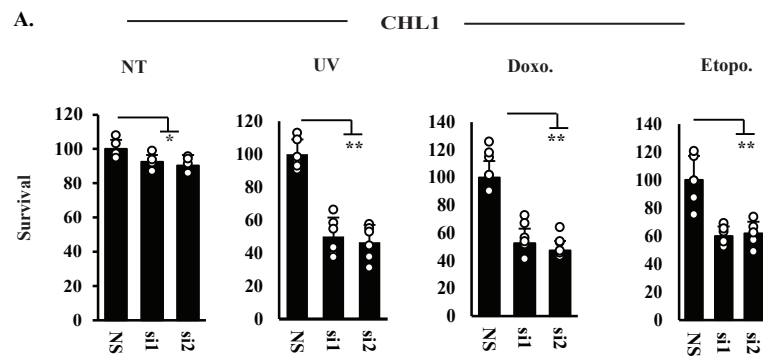

B.

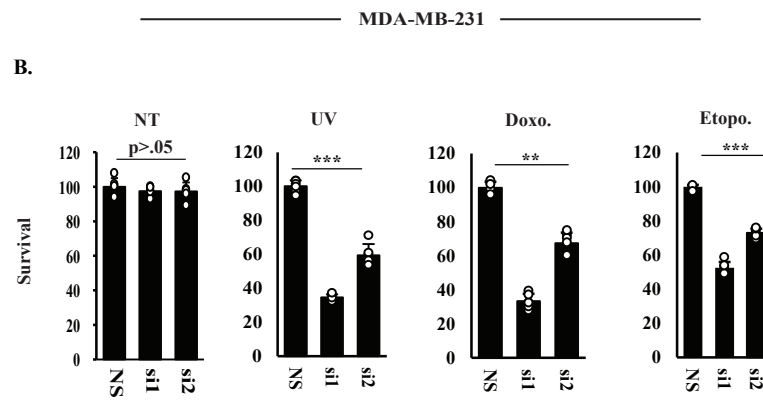

C.

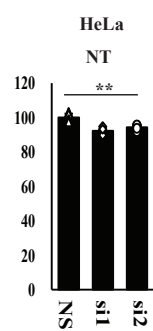

Supplement: Supplementary file 2 — Supplement Figure 1 [file 41388_2024_3021_MOESM2_ESM.pdf]
